# Apical dendrite degeneration, a novel cellular pathology for Betz cells in ALS

**DOI:** 10.1038/srep41765

**Published:** 2017-02-06

**Authors:** Barış Genç, Javier H. Jara, Amiko K. B. Lagrimas, Peter Pytel, Raymond P. Roos, M. Marsel Mesulam, Changiz Geula, Eileen H. Bigio, P. Hande Özdinler

**Affiliations:** 1 Department of Neurology and Clinical Neurological Sciences, Northwestern University, Feinberg School of Medicine, Chicago, IL, 60611, USA; 2 Department of Pathology, University of Chicago Medical Center, Chicago, IL, 60637, USA; 3 Department of Neurology, University of Chicago Medical Center, Chicago, IL, 60637, USA; 4 Cognitive Neurology and Alzheimer’s Disease Center, Northwestern University, Chicago, IL, 60611, USA; 5 Robert H. Lurie Cancer Center, Northwestern University, Chicago, IL, 60611, USA

## Abstract

Apical dendrites of Betz cells are important sites for the integration of cortical input, however their health has not been fully assessed in ALS patients. We investigated the primary motor cortices isolated from post-mortem normal control subjects, patients with familial ALS (fALS), sporadic ALS (sALS), ALS with frontotemporal dementia (FTD-ALS), and Alzheimer’s disease (AD), and found profound apical dendrite degeneration of Betz cells in both fALS and sALS, as well as FTD-ALS patients. In contrast, Betz cells of AD patients and normal controls retain cellular integrity in the motor cortex, and CA1 pyramidal neurons show abnormalities predominantly within their soma, rather than the apical dendrite. In line with extensive vacuolation and cytoarchitectural disintegration, the numbers of synapses were also significantly reduced only in ALS patients. Our findings indicate apical dendrite degeneration as a novel cellular pathology that distinguishes ALS and further support the importance of cortical dysfunction for disease pathology.

Upper motor neurons (Betz cells; corticospinal motor neurons (CSMN) in mice) are important components of the motor neuron circuitry^1^ because of their ability to initiate and modulate voluntary movement. Even though Betz cell degeneration^2^,^3^,^4^,^5^,^6^,^7^,^8^,^9^,^10^,^11^,^12^,^13^,^14^,^15^,^16^ is accepted as a defining characteristic of amyotrophic lateral sclerosis (ALS)^12^,^17^,^18^,^19^,^20^,^21^, the mode and the extent of their cellular pathology is actively debated^22^,^23^,^24^.

Previous studies suggested directional degeneration from neuromuscular junction towards cerebral cortex^22^, diminishing the importance of Betz cells as an important contributor to disease pathology. Today, however, multiple evidence suggests upper motor neurons as a significant contributor to disease pathology^17^,^20^,^25^,^26^. Increased cortical hyperexcitability observed prior to disease symptom manifestation in ALS patients, suggests that Betz cells display early neuronal pathology, and that their cortical connections are impaired prior to disease onset^17^,^18^,^20^,^21^.

We find that Betz cells of patients with familial (fALS) and sporadic ALS (sALS) as well as patients with ALS and frontotemporal dementia (FTD-ALS), display significant cellular pathology, particularly vacuoles along their apical dendrites, recapitulating the findings in diseased CSMN^17^,^27^,^28^,^29^,^30^,^31^,^32^,^33^,^34^. Such defects are not observed in the motor cortex of Alzheimer’s disease (AD) patients or controls. Likewise, CA1 pyramidal neurons in AD patients do not display apical dendrite abnormalities even though they degenerate, suggesting that dendrite pathology is mainly observed in Betz cells of ALS, and that cortical connectivity defects could be an early and an important contributor to ALS pathology in patients.

## Results

**Figure 1:**
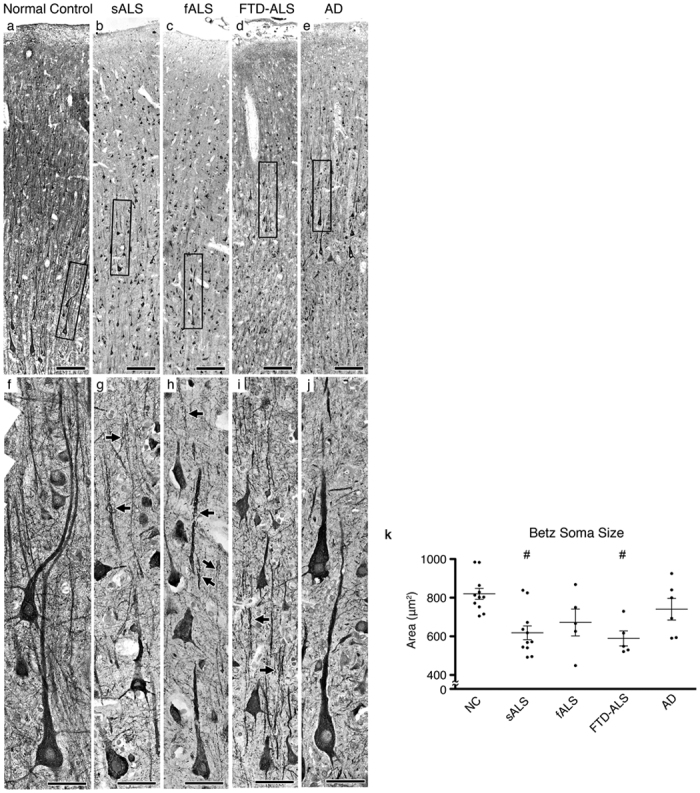
Betz cells are present in layer V of the motor cortex. (**a**–**e**) Representative image of motor cortices isolated from normal control subjects (**a**), or sALS (**b**), fALS (**c**), FTD-ALS (**d**), and AD (**e**) patients. Betz cells are identified by their presence in layer V of the motor cortex, their large pyramidal soma and prominent apical dendrite (box). Boxed areas are enlarged below (**f**–**j**). Representative images of Betz cells located in layer V of the motor cortex in normal controls (**f**), or sALS (**g**), fALS (**h**), FTD-ALS (**i**), and AD (**j**) patients. Arrows point to apical dendrites containing vacuoles. (**k**) Scatter plots represent mean area ± SEM of Betz cells in all cases studied. Normal controls versus diseased cases studied ^#^*P* < 0.05. One-way ANOVA (Kruskal-Wallis test) with *post hoc* non-parametric Dunn’s multiple comparison test. Abbreviations: NC: normal control, sALS = sporadic ALS, fALS = familial ALS, FTD-ALS = ALS with frontotemporal dementia, and AD = Alzheimer’s disease. (**a**–**e**) Scale bar = 200 μm, (**f**–**j**) scale bar = 50 μm.

Betz cells are the largest projection neurons of the cerebral cortex, and are distinguished by their location, large soma and prominent apical dendrite. The health and the integrity of their apical dendrites are paramount for proper modulation both by local and long distance projection neurons. In order to assess neuronal integrity of Betz cells, primary motor cortices were isolated and investigated from post-mortem brains of normal controls (*n* = 11) and patients with fALS (*n* = 5), sALS (*n* = 10), FTD-ALS (*n* = 5), and AD (*n* = 6). Betz cells were distinguished from other cortical neurons by their large size, and location within layer V of the motor cortex (Fig. 1a,f)^5^,^16^.

Betz cells soma size shrinkage was apparent in layer V of the motor cortex in patients with sALS (Fig. 1b,g), fALS (Fig. 1c,h), and FTD-ALS (Fig. 1d,i), as observed previously^6^,^8^,^10^. Mean areas of Betz cells in normal controls were 820 ± 28 μm^2^ (*n* = 57 neurons; Fig. 1k) and 671 ± 70 μm^2^, (*n* = 46 neurons) in fALS patients. The average Betz cell soma area was significantly reduced in sALS (619 ± 40 μm^2^, *n* = 75 neurons, adjusted *P* value = 0.0196; Fig. 1k)) and FTD-ALS (589 ± 3 9 μm^2^, *n* = 34 neurons, adjusted *P* value = 0.0297; Fig. 1k)) patients. However, the mean areas of Betz cells in AD patients (740 ± 56 μm^2^, *n* = 37 neurons) were comparable to that of normal controls. Interestingly though, close examination (Fig. 1f–j) suggested prominent neuronal defects, especially along the apical dendrites. There were numerous apical dendrites filled with vacuoles, consistent with a severe cytoarchitectural defect and potentially a site of neuronal degeneration. In contrast, Betz cells in AD patients (Fig. 1e,j) displayed healthy morphology and apical dendrites were similar to those of normal controls.

Since it is not always possible to have a cell body and the apical dendrite intact on the same plane on a 4 μm thick cortical section, we imaged apical dendrites that stood out because of their size, thickness, tufted appearance and directional projection. Betz cells are the largest pyramidal neurons present in layer V of the motor cortex, and their apical dendrites were more easily distinguished in layer IV, but the neuronal identity of apical dendrites in layer II/III was not possible to discern. Therefore, in an effort to specifically assess the integrity of Betz cell apical dendrites in greater detail, we focused our attention on layer IV–V, and not layer II/III.

**Figure 2:**
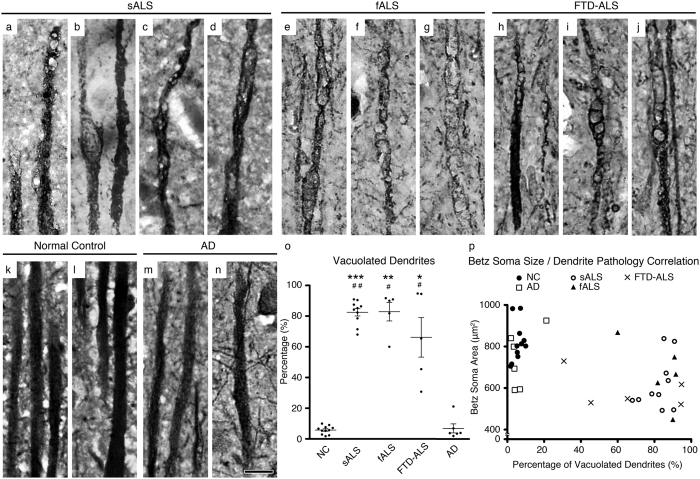
Apical dendrites are filled with vacuoles and disintegrate only in ALS. (**a**–**n**) Representative images of prominent apical dendrites that are located in layer IV of the motor cortex in sALS (**a**–**d**), fALS (**e**–**g**), and FTD-ALS (**h**–**j**) patients and normal control subjects (**k**–**l**). Apical dendrites that contain vacuoles and show signs of degeneration are marked with arrows. In contrast to ALS, apical dendrites in AD patients (**m**,**n**) are similar to normal controls and lack signs of degeneration. (**o**) Scatter plots represent mean percentage ± SEM of apical dendrites containing vacuoles in all diseased cases studied. Normal controls versus diseased cases studied *P < 0.05, **P < 0.01, ***P < 0.001. AD versus diseased cases studied ^#^*P* < 0.05, ^##^*P* < 0.01. One-way ANOVA (Kruskal-Wallis test) with *post hoc* non-parametric Dunn’s multiple comparison test. (**p**) Scatter plot representing average Betz soma area plotted against percentage of apical dendrites containing vacuoles for each subject. Abbreviations: NC: normal control, sALS = sporadic ALS, fALS = familial ALS, FTD-ALS = frontotemporal dementia and ALS, and AD = Alzheimer’s disease. Scale bar = 10 μm.

There were numerous structural defects in comparison with normal controls, including large vacuoles or vacuoles with different shapes and sizes, which severely impaired the integrity of the cytoarchitecture within the apical dendrites of large pyramidal neurons in the motor cortex of patients with sALS (82 ± 2%, *n* = 206 apical dendrites, adjusted *P* value = 0.0048; Fig. 2a–d,o), fALS (83 ± 6%, *n* = 98 apical dendrites, adjusted *P* value = 0.0004; Fig. 2e–g,o), and FTD- ALS (66 ± 13%, *n* = 142 apical dendrites, adjusted *P* value = 0.0273; Fig. 2h–j,o). In some cases, the apical dendrites displayed profound disintegration, which appeared to be greatest distally (Fig. 2h). In contrast, apical dendrites appeared healthy in normal controls (6 ± 1%, *n* = 288 apical dendrites; Fig. 2k,l,o) and patients with AD (7 ± 3%, *n* = 246 apical dendrites; Fig. 2m–o). Since, dendritic spines could not be observed in any of the apical dendrites (*n* = 923), including normal controls, with the Map2 immunocytochemistry, it was not possible to assess spine health or reduction in spine numbers. Initial quantitative assessment revealed that cytoarchitectural defects were significant in the motor cortex of patients with sALS, fALS, and FTD-ALS, whereas the motor cortex from patients with AD was comparable with that of normal controls. In an unbiased scatter plot of Betz soma size versus percentage of vacuolated apical dendrites (Fig. 2p), AD patients clustered with normal controls (large Betz soma areas and low percentage of vacuolated dendrites) whereas patients with sALS and fALS clustered together (smaller Betz soma areas and high percentage of vacuolated dendrites), displaying a negative correlation between Betz soma size and dendritic pathology (Pearson’s correlation coefficient *R* = −0.558; Fig. 2p).

**Figure 3:**
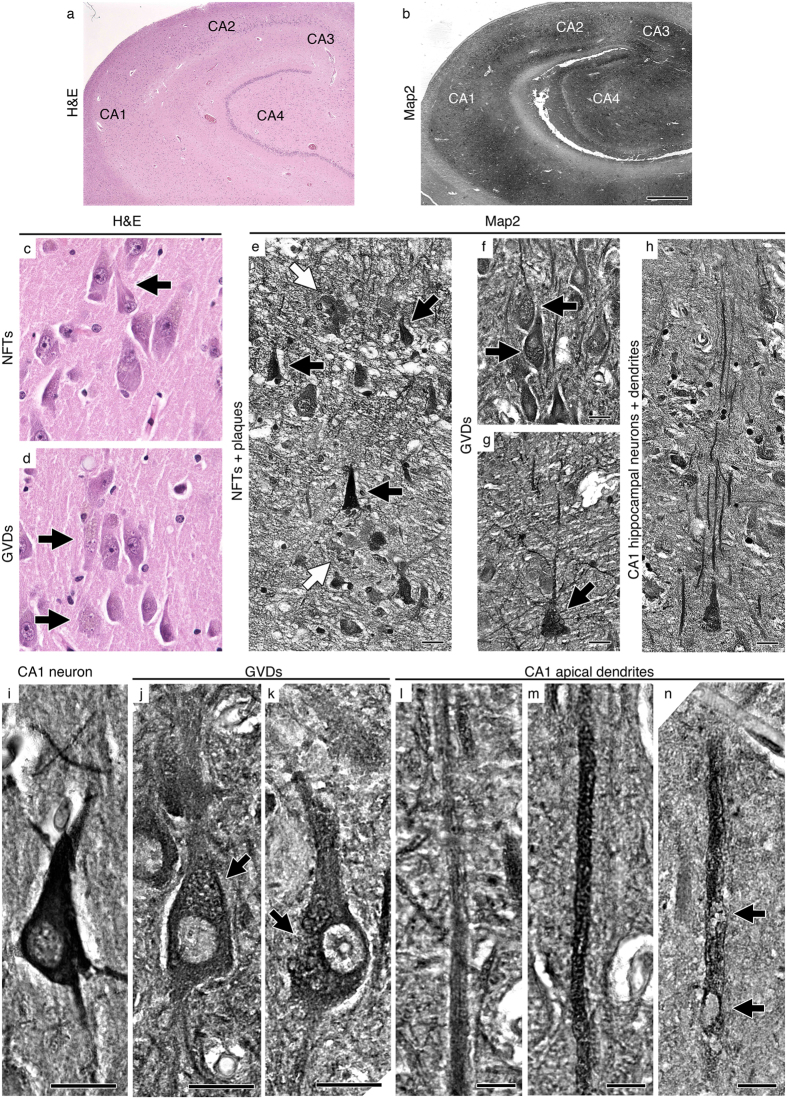
Hippocampal neuron cell bodies, but not dendrites are filled with vacuoles in AD. (**a**) Representative image of H&E stained AD hippocampus. (**b**) Representative image of Map2 immunohistochemistry in AD hippocampus. (**c**,**d**) Hippocampal neurons as observed in an H&E stained slide. Arrows point to NFTs (**c**) and GVD (**d**). (**e**–**h**) Hippocampal neurons as observed by Map2 immunohistochemistry. Black arrows point to NFTs (**e**) and GVD (**f**,**g**), white arrows point to plaques. (**i**–**k**) Representative images of normal (**i**) and GVD (**j**,**k**) pyramidal neurons in AD CA1. Arrows point to vacuoles. (**l**–**n**) Representative images of normal (**l**,**m**) and vacuolated (**n**) apical dendrites in AD CA1. Arrows point to vacuoles. Abbreviations: H&E = hematoxylin and eosin, CA = cornu ammonis, NFT = neurofibrillary tangle, GVD = granulovacuolar degeneration, and AD = Alzheimer’s disease. (**b**) Scale bar = 1mm, (**e**–**k**) scale bar = 20 μm, (**l**–**n**). Scale bar = 10 μm.

To investigate whether the observed apical dendrite degeneration with vacuoles is pathology unique to ALS motor cortex or a broad phenomenon observed in other neurodegenerative diseases, we assessed neuronal integrity of pyramidal neurons of the CA1 hippocampus in AD patients (Fig. 3a,b). This region is known to have the highest neuron loss in AD^35^. The main morphologic abnormalities in the AD hippocampus are neurofibrillary tangles (NFT) and granulovacuolar degeneration (GVD)^36^. Hematoxylin and eosin (H&E) staining (Fig. 3c,d) revealed NFT and GVD in the CA1 region, as expected. Map2 immunocytochemistry also helped reveal GVD (Fig. 3e–g), as well as pyramidal neurons and their apical dendrites (Fig. 3h). NFT were darkly immunolabeled by Map2 in the CA1 region of all 5 AD patients, whereas 3 out of 5 patients displayed massive GVD-related vacuoles especially in cell bodies (Fig. 3i–k). Even though there was variation from patient to patient on the number and the extent of GVD-related somatic vacuoles, none of the patients had vacuoles on the apical dendrites of the CA1 region neurons (Fig. 3l–n). Despite severe neurodegeneration in the CA1 region, apical dendrites mostly appeared healthy, and only a very few dendrites contained vacuoles (6 ± 1%, *n* = 290 apical dendrites). In contrast, the degenerating neurons in the CA1 region of hippocampus in AD patients mostly suffered from dystrophic neurites associated with plaques, as well as NFT and GVD-related defects inside their soma, as extensively reported in the past^35^,^36^. These findings indicate a significant difference between two important neuron populations that become vulnerable in ALS and AD, and suggest that the cellular basis of their vulnerability and mode of degeneration may differ.

**Figure 4:**
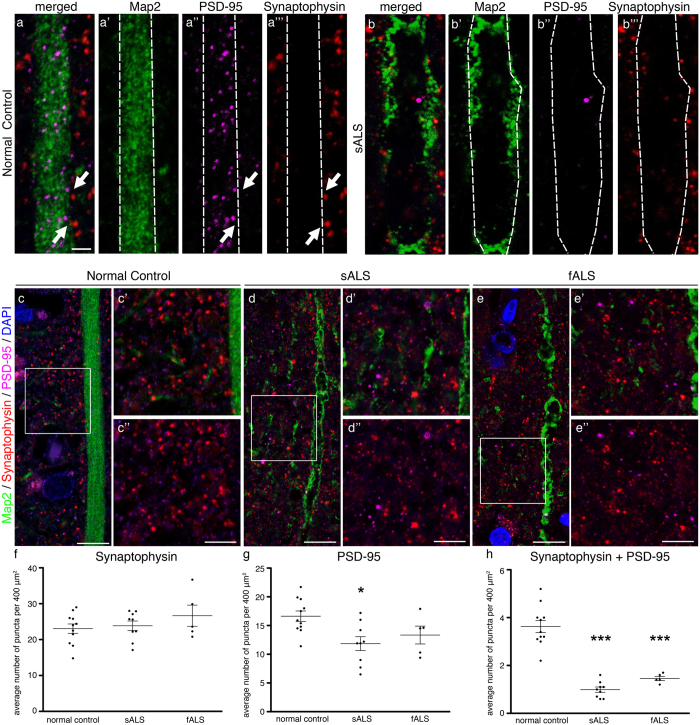
Post-synaptic densities are decreased in ALS motor cortex. (**a**,**b**) Representative images of Map2 (apical dendrites), synaptophysin (pre-synaptic terminals), and PSD-95 (post-synaptic zone) immunocytochemistry in normal control (**a**), and sALS (**b**) motor cortex. Dashed lines represent apical dendrite boundaries. Arrows point to pairs of PSD-95 puncta inside the dendrite and synaptophysin puncta outside the dendrite, within close proximity of each other. (**c**–**e**) Representative areas used for quantification of synaptic puncta density in normal control (**c**), sALS (**d**) and fALS (**e**) motor cortices are boxed, and enlarged (c’,d’,e’). Green channel is removed for easier observation of synaptic staining (c”,d”,e”). (**f**–**h**). Scatter plots represent mean average ± SEM number of puncta per 400 μm^2^ (20 μm × 20 μm) area quantified in all cases studied. Normal controls versus diseased cases studied *P < 0.05, ***P < 0.001. One-way ANOVA (Kruskal-Wallis test) with *post hoc* non-parametric Dunn’s multiple comparison test. Abbreviations: NC: normal control, sALS = sporadic ALS, fALS = familial ALS. (**a**,**b**) scale bar = 2 μm, (**c**–**e**) scale bar = 10 μm, (c’,c”,d’,d”,e’,e”) scale bar = 5 μm.

Apical dendrites of Betz cells are especially important for receiving cortical input. To investigate whether the apical dendrite defects observed in ALS subjects affect synaptic integrity and cortical connectivity, we quantified the number of pre- and post-synaptic densities at the site of the apical dendrite, using synaptophysin and PSD-95 immunocytochemistry, respectively (Fig. 4). Along the Map2+ apical dendrites, post-synaptic puncta (PSD-95+) were mostly observed within the dendrite (Fig. 4a”,b”), and pre-synaptic puncta (synaptophysin+) were located outside dendrite boundaries (Fig. 4a”’,b”’; dashed lines define the borders of the apical dendrite). Although some presynaptic and postsynaptic puncta could be observed in close proximity (i.e. 2 μm) along the Betz cell dendrite (Fig. 4a, arrows), it was not possible to assess or quantify the number of synapses received by a given Betz cell as it was not possible to visualize the dendritic spines of Betz cells with clarity and precision. Since numerous neurons, such as long distance projection, local circuitry and interneurons cumulatively contribute to Betz cell modulation especially at the site of apical dendrites^37^, we took a broader perspective to investigate if average density of synaptic puncta^38^ were altered near the close vicinity of the apical dendrite (i.e. 20 μm × 20 μm boxed area), where most direct and indirect connections that ultimately affect Betz cell modulation are made. The average numbers of pre-synaptic densities in normal controls (23 ± 1.3, *n* = 100 apical dendrites), as well as sALS (24 ± 1.3, *n* = 90 apical dendrites) and fALS (27 ± 3, *n* = 50 apical dendrites) patients were comparable (Fig. 4f). In contrast, the average numbers of post-synaptic densities were reduced. The numbers of post-synaptic densities in normal controls (17 ± 1, *n* = 100 apical dendrites) were significantly higher than that of sALS patients (12 ± 1.2, *n* = 90 apical dendrites, adjusted *P* value = 0.0114; Fig. 4g). Even though the numbers of post- synaptic densities were comparable between normal controls and fALS (13 ± 2, *n* = 50 apical dendrites; Fig. 4g), the average numbers of puncta co-labeled with both synaptophysin and PSD-95, a sign of active synapse, was significantly reduced in both sALS (1 ± 0.1, *n* = 90 apical dendrites, adjusted *P* value < 0.0001) and fALS patients (1.5 ± 0.1, *n* = 50 apical dendrites, adjusted *P* value < 0.0001), when compared to normal controls (4 ± 0.3, *n* = 100 apical dendrites; Fig. 4h).

These findings indicate that at the close vicinity of the vacuolated and disintegrated apical dendrites the number of healthy and functional synapses were reduced, and this indeed could be the underlying cause for selective neuronal vulnerability of a neuron population that is dependent on cortical modulation and input for proper function.

## Discussion

In neurodegenerative diseases distinct neuron populations display early vulnerability and undergo progressive degeneration. The cellular and molecular basis of this selective vulnerability and cellular pathology that occurs early in the disease, are not fully understood. However, focusing attention to the neurons that become vulnerable may begin to reveal the mode of disease initiation and progression.

Betz cells are one of the most important cortical components of motor neuron circuitry, and their degeneration is a hallmark of ALS, which is characterized by the progressive loss of both upper and lower motor neurons. Betz cells are the largest excitatory neurons of the cerebral cortex with volumes larger than the volumes of other pyramidal cells by a factor of twenty^39^. As one of the largest projection neurons in our CNS, their axon can be more than a meter long in some people. During disease, however, the soma size of Betz cells is significantly reduced^6^,^8^,^10^, and axons within the corticospinal tract degenerate^8^,^12^,^15^. Even though the apical dendrite is the active site for Betz cell modulation, its health and integrity has not been investigated in detail. Our data confirms reduction of Betz soma size in patients with sALS and FTD-ALS, however due to small sample size (*n* = 5), and broader variation among fALS patients, the reduction did not reach statistical significance. Interestingly though, one of the patients who had relatively large Betz cells (868 ± 229 μm^2^ and only 60% vacuolated apical dendrites; Table 1, case #22) and predominantly LMN pathology, carried the SOD1I113T mutation, which was previously reported to show low penetrance and variable clinical manifestations^9^. In contrast, patient with SOD1G93A mutation displayed one of the smallest Betz soma with highest percentage of vacuolation (448 ± 95 μm^2^ and 90% vacuolated apical dendrites; Table 1, case #24).

One potential reason for the lack of detailed cortical studies could be the long-lasting prominence of “dying-back” hypothesis^22^, which postulates that degeneration occurs in a directional fashion, progressing from neuromuscular junction towards cerebral cortex. Based on this view, the Betz cells would be the last site of neuronal pathology and would not contribute to disease. For many years this hypothesis underestimated the importance of early cerebral dysfunction in ALS.

Building evidence now suggests early cortical dysfunction in ALS patients and that Betz cells can be a cellular target for future therapies^17^,^18^,^19^,^20^,^25^,^26^. Threshold tracking transcranial magnetic stimulation with indices including short interval intracortical inhibition revealed a wave of early hyperexcitation in the motor cortex of ALS patients, detected even prior to symptom onset^18^,^20^,^21^. Cortical hyperexcitability has been demonstrated to be an early^40^,^41^, selective^20^,^26^,^42^, and powerful^21^ biomarker for diagnosis of ALS. In addition, cortical defects and axonal degeneration within the corticospinal tract were assessed using non-invasive proton magnetic resonance spectroscopy in ALS patients revealing early signs of cerebral cortex dysfunction^19^. To assess potential changes and cellular defects along the apical dendrites of upper motor neurons, our study closely investigates large pyramidal neurons located in layer V of the motor cortex in fALS, sALS, FTD-ALS as well as AD patients and controls. We find massive apical dendrite degeneration in the motor cortex of a broad spectrum of ALS patients, and this phenomenon is not observed in AD patients or the normal controls. Pyramidal neurons of the CA1 region of the hippocampus are mainly affected in AD, but different from Betz cells in ALS patients their cellular pathology is restricted to soma and their apical dendrites retain their integrity.

It is feasible to think that neurons become vulnerable and display early signs of degeneration when they fail to perform their key function. The Betz cells act as the “spokesperson” of the cerebral cortex as they receive, integrate, translate and transmit cerebral cortex’s input towards spinal cord targets to initiate and modulate voluntary movement and motor function. Since the apical dendrite is the main site of synaptic integration, it is possible that a dysfunctional apical dendrite would selectively impair the ability of Betz cells to be modulated, and therefore conveying cerebral cortex’s message to the spinal cord targets would be impaired, leading to their selective vulnerability.

Spine loss is one of the common underlying causes of neuronal vulnerability and it is observed in many degenerating neurons in different diseases^43^,^44^. Synapse loss^45^,^46^ and reduced dendritic complexity and spine pathology^47^ have long been recognized as important contributors to pathology of AD and a broad spectrum of other neurodegenerative diseases^43^,^44^. Interestingly, early studies with Golgi staining revealed spine loss in large pyramidal neurons located in layer V of the motor cortex in post-mortem samples obtained from ALS patients^5^,^16^.

In addition, in ALS mouse models, CSMN that become diseased due to many different underlying causes, such as SOD1 mutation^32^, TDP43 pathology^30^, absence of Alsin^29^, and increased ER-stress^31^ also show early and profound spine loss, especially along the apical dendrite. Spine loss as early as P21 in the hSOD1^G93A^ mice^27^,^28^ was reported, adding to our previous findings demonstrating early cytoarchitectural defects mainly in the apical dendrites of CSMN in this ALS model. Using a rat model of hSOD1^G93A^, a recent study demonstrated that reducing mutant SOD1 in the motor cortex was enough to improve the health of spinal motor neurons and that of neuromuscular junction^48^, questioning the feasibility of “dying-back” hypothesis, and suggesting that upper motor neurons could indeed be cellular targets for ALS^49^. Synaptopathies develop due to defects either in the pre-synaptic or post- synaptic terminals, and sometimes both are affected.

Our studies suggest that the cortical connectivity at the site of Betz cell apical dendrite is impaired. Since we are faced with the technical limitation of visualization and assessing individual spines, future studies are required for detailed and precise analysis of spines, potentially with 3D reconstruction^50^ and imaging with electron microscopy. It is our hope that this report will initiate future studies in which the health and integrity of Betz cell apical dendrites will be closely investigated with respect to cortical hyperexcitability, physiological changes in patients based on site of onset and mode of disease progression in patients. Our current studies reveal cellular defects that are mainly observed in the apical dendrites of large pyramidal neurons in the motor cortex, revealing a potential cortical connectivity defects in a broad spectrum of ALS patients, and suggesting that apical dendrite disintegration and loss of active synapses at the site of apical dendrites of Betz cells could contribute to selective upper motor neuron vulnerability and disease pathology in ALS.

## Methods

### Post-mortem human brain samples

Postmortem human tissue was obtained from the University of Chicago and Northwestern University according to protocols approved by the institutional review boards at each institution. All experiments were performed in accordance with relevant guidelines and regulations of both institutions. All methods were carried out in accordance with relevant guidelines and regulations, and all experimental protocols were approved by the institutional review boards at each institution. Informed consent was obtained from all subjects and clinical records were available for every subject. All patients were examined by neurologists and all autopsy tissue was examined by neuropathologists. Brains were fixed either in 10% neutral buffered formalin for two weeks or 4% paraformaldehyde at 4 °C for 30 hours, and sections were paraffin embedded. Areas of the primary motor cortex, M1, from BA4, in the region of the hand, as determined by the “motor homunculus” were retrieved, 4 μm thick serial sections were cut, mounted on a charged glass slide (Fisher Scientific, Pittsburg, PA), and used for immunocytochemical analyses. In this study, motor cortex isolated from 11 normal control subjects with no neurologic disease, 5 fALS patients with mutations in SOD1, 10 sALS patients without a family history of ALS, 5 FTD-ALS patients, and 6 AD patients, and hippocampus isolated from 5 AD patients were included. The age, sex, and diagnosis of patients are documented in Table 1.

### Immunocytochemistry

Slides were baked for 60 min at 60 °C, deparaffinized with xylene for 5 min, rehydrated in ethanol (100%, 95%, 70%, and 50%). For antigen retrieval, slides were immersed in 10 mM sodium citrate and subjected to high heat and pressure for 20 min. After cooling, slides were rinsed with PBS for 10 min and blocked with 0.5% bovine albumin serum, 0.1% Triton X-100, and 2% fetal bovine serum in PBS for 30 min, and incubated overnight at 4 °C with a mix of primary antibodies MAP2 (Millipore, cat# AB5622, 1:200, Temecula, CA), synaptophysin (Millipore, cat# MAB368, 1:200, Temecula, CA), PSD-95 (abcam, cat# ab12093, 1:200, Cambridge, MA). After PBS rinses, slides were incubated with AlexaFluor conjugated secondary antibodies diluted in the blocking solution (donkey anti rabbit AF488, cat# A21206, 1:500; donkey anti mouse AF555, cat# A31570, 1:500; donkey anti goat AF647, cat# A21447, 1:500, Life Technologies, Carlsbad, CA) for 2 hours at room temperature. Slides were rinsed in PBS, counterstained with DAPI, lipofuscin autofluoresence was quenched with True Black (Biotum, cat# 23007, Hayward CA) according to manufacturer’s instructions, and coverslipped with Fluoromount G (Electron Microscopy Sciences, cat# 17984-25, Hatfield, PA).

### Imaging and quantification

Betz cells were identified based on their location in layer V of the motor cortex and their large pyramidal cell body, and long, thick apical dendrite. All Betz cells with a full soma and a prominent apical dendrite in layer V, and apical dendrites in layer IV distinguished by their size and thickness, were imaged using Nikon SMZ1500 and Nikon Eclipse TE2000-E microscopes equipped with Intensilight C-HGFI (Nikon Inc., Melville, NY). Light images were acquired using a Ds-Fi1 camera (Nikon Inc., Melville, NY) using a 63X objective. Fluorescently labeled sections were imaged using a Zeiss LSM 880 confocal microscope (Carl Zeiss Microscopy LLC, Thornwood, NY). The percentage of apical dendrites that displayed cytoarchitectural defects, signs of disintegration and vacuolation was quantified for each subject and disease. Betz cells areas were determined using NIS Elements software (Nikon Inc., Melville, NY) with images taken using a 20X objective to determine differences in soma size. Betz cells with areas >300 μm^2^ in the layer V with a visible nucleus and proximal apical dendrite were assessed for each subject (a minimum of *n* = 6 Betz cells per subject). For quantification of synapses, 0.9 μm thick optical sections of confocal images from Map2/Synaptophysin/PSD-95 stained brain sections were used. The number of pre- and post-synaptic puncta were counted in a 20 μm by 20 μm box drawn adjacent to apical dendrites from 10 neurons per subject.

### Statistical analysis

All statistical analyses were performed using Prism software (version 6; Graphpad Software Inc., La Jolla, CA). Statistically significant differences were determined after one-way ANOVA (Kruskal-Wallis test) with *post hoc* non- parametric Dunn’s multiple comparison test. Statistically significant differences are reported with adjusted *P* values, and data is expressed as the mean ± SEM. Pearson’s correlation coefficient was calculated using Excel (Microsoft, Redmond WA).

## Additional Information

**How to cite this article:** Genç, B. *et al*. Apical dendrite degeneration, a novel cellular pathology for Betz cells in ALS. *Sci. Rep.* **7**, 41765; doi: 10.1038/srep41765 (2017).

**Publisher's note:** Springer Nature remains neutral with regard to jurisdictional claims in published maps and institutional affiliations.

## Figures and Tables

**Table 1:** Post-mortem motor cortex samples included in this study.

Case	Age, y	Sex	Clinical diagnosis	UMN/LMN predominant	Postmortem interval, h
1	52	M	normal control	—	27
2	58	F	normal control	—	24
3	45	F	normal control	—	15
4	60	M	normal control	—	19
5	59	M	normal control	—	12
6	64	F	normal control	—	6
7	67	M	normal control	—	5
8	75	F	normal control	—	17
9	88	M	normal control	—	27
10	100	F	normal control	—	14
11	87	M	normal control	—	16
12	55	M	sporadic ALS	LMN > UMN	20
13	68	F	sporadic ALS	LMN > UMN	17
14	66	M	sporadic ALS	LMN > UMN	18
15	62	F	sporadic ALS	LMN > UMN	7
16	68	F	sporadic ALS	LMN > UMN	19
17	49	F	sporadic ALS	LMN > UMN	15
18	61	M	sporadic ALS	LMN > UMN	17
19	61	M	sporadic ALS	LMN > UMN	13
20	64	F	sporadic ALS	UMN = LMN	9
21	66	F	sporadic ALS	LMN > UMN	12
22	79	F	familial ALS (SOD1 I113T)	LMN > UMN	24
23	66	M	familial ALS (SOD1 E100G)	UMN = LMN	4
24	64	M	familial ALS (SOD1 G93A)	LMN > UMN	18
25	53	M	familial ALS (SOD1 I113T)	LMN > UMN	7
26	50	F	familial ALS (SOD1 V148G)	LMN > UMN	4
27	61	M	FTD and ALS	UMN = LMN	4
28	61	M	FTD and ALS	LMN > UMN	8
29	64	F	FTD and ALS	UMN = LMN	6
30	58	M	FTD and ALS	LMN > UMN	8
31	59	F	FTD and ALS	LMN > UMN	21
32	69	M	AD	—	8
33	64	F	AD	—	22
34	59	M	AD	—	26
35	84	M	AD	—	23
36	83	M	AD	—	9
37	81	M	AD	—	19
38*	72	M	AD	—	14
39*	62	M	AD	—	21
40*	55	F	AD	—	28
41*	79	F	AD	—	18
42*	66	M	AD	—	18

Case numbers, age, sex and diagnosis of patients, UMN/LMN predominance and postmortem interval are included. LMN > UMN: lower motor neuron pathology preceded upper motor neuron pathology; UMN = LMN: both upper and lower motor neuron pathology observed during time of diagnosis. Abbreviations: FTD = frontotemporal dementia; AD = Alzheimer’s disease; UMN = upper motor neuron; LMN = lower motor neuron; *hippocampus.
